# PROTAC for Bruton’s tyrosine kinase degradation alleviates inflammation in autoimmune diseases

**DOI:** 10.1038/s41421-024-00711-x

**Published:** 2024-08-06

**Authors:** Can Zhu, Zimo Yang, Yuxiao Zhang, Zhenjun Li, Guangchen Li, Bing Yang, Na Kang, Jingwen Wang, Yonghui Sun, Ning Ding, Yu Rao, Wanli Liu

**Affiliations:** 1 State Key Laboratory of Membrane Biology, School of Life Sciences, Tsinghua-Peking Center for Life Sciences, Institute for Immunology, Ministry of Education Key Laboratory of Protein Sciences, Beijing Key Lab for Immunological Research on Chronic Diseases, Beijing Tsinghua Changgeng Hospital, Tsinghua University, Beijing, China; 2 The First Affiliated Hospital of Anhui Medical University and Institute of Clinical Immunology, Anhui Medical University, Hefei, Anhui China; 3 MOE Key Laboratory of Protein Sciences, School of Pharmaceutical Sciences, Ministry of Education Key Laboratory of Bioorganic Phosphorus Chemistry and Chemical Biology, Tsinghua University, Beijing, China; 4 Key Laboratory of Carcinogenesis and Translational Research (Ministry of Education), Department of Lymphoma, Peking University Cancer Hospital & Institute, Beijing, China

**Keywords:** Autoimmunity, Proteolysis

Dear Editor,

Proteolysis-targeting chimera (PROTAC) is a newly emerging strategy that harnesses the ubiquitin–proteasome system (UPS) to knock down target proteins for novel therapies^1^. This approach has rapidly garnered attention in both academia and industry fields. We designed the first PROTAC degrader to target Bruton’s tyrosine kinase (BTK), a key protein for B-cell survival and function, for the purpose of treating mutated BTK C481S- induced Ibrutinib-resistant B-cell malignancies^2^. We then developed a new generation of BTK degrader L18I through intensive ligand and linker optimizations, exhibiting improved solubility and superior efficiency in degrading BTK^3^. L18I exhibits excellent degradability towards different mutated forms of BTK proteins in human B-cell-derived non-Hodgkin lymphoma, and also inhibits the growth of activated human B-cell-like diffuse large B-cell lymphoma tumor cell in xenograft mice without obvious side effects^3^. Apart from these BTK-mediated B-cell tumor studies^4^, numerous evidence has highlighted the significant involvement of BTK dysfunction in autoimmune diseases^5^. Indeed, BTK inhibitors have been recently investigated for the treatment of autoimmune diseases, however, there are both successes and failures in clinical trials due to clinical efficacy and safety concerns^5^. Although PROTACs for BTK degradation have been shown minimal off-target effects as demonstrated in this report (Supplementary Fig. S1a–c), and in our literature^2,3^, the application of BTK-targeting PROTACs in autoimmune diseases is lack. We studied the effects of the BTK degrader L18I on autoimmune diseases by using BM12 splenocytes-induced lupus disease model and the pristane-induced diffuse alveolar hemorrhage (DAH) disease model.

**Fig. 1:**
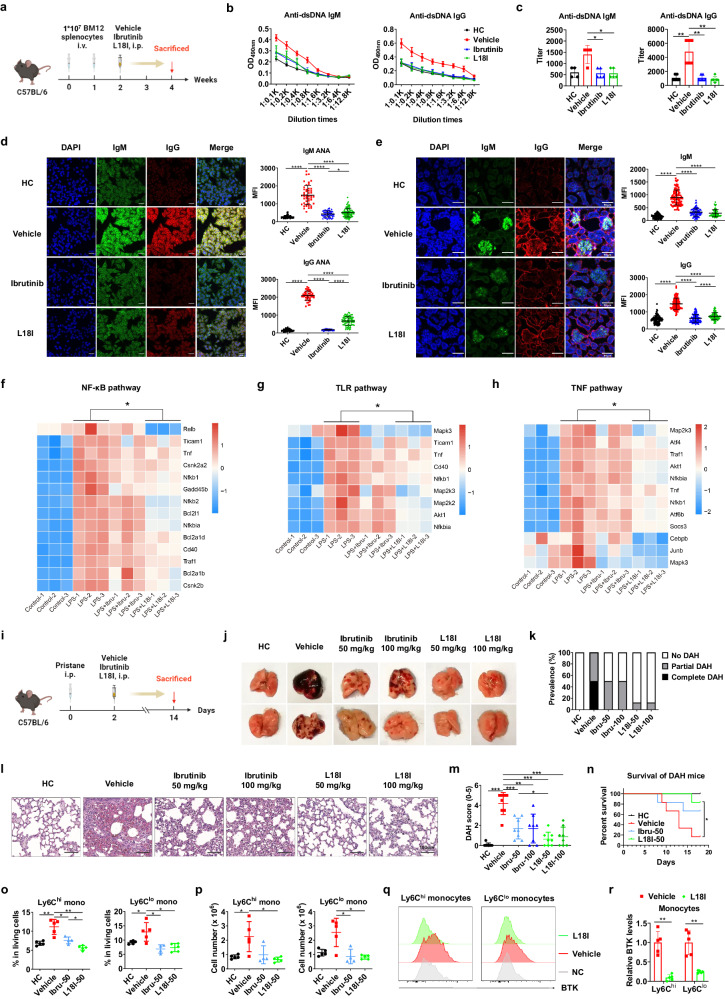
BTK degrader L18I relieved the symptoms of BM12-induced lupus and pristane-induced DAH mouse models. **a** Diagrammatic representation of adoptively transferring BM12 splenocytes to C57BL/6 mice to induce lupus-like model. Vehicle, Ibrutinib, and L18I treatment (50 mg/kg, i.p., twice a day, *n* = 6) began at the second week of induction and continued for two weeks. **b**–**d** OD_490nm_ of serum anti-dsDNA IgM and IgG (**b**), and their titers (**c**), ANA IgM and IgG antibodies (**d**) were detected from **a**. **e** Immunofluorescence and mean fluorescence intensity (MFI) quantification of deposited IgM and IgG antigen–antibody immune complexes in the glomeruli from **a**. **f**–**h** Primary B-cells treated with DMSO, Ibrutinib (200 nM) and L18I (200 nM) were stimulated by LPS (1 μg/mL) for 12 h in vitro, and the NF-κB pathway (**f**), TLR pathway (**g**), and TNF pathway (**h**) expression profile were presented using RNA-seq analysis. **i** Diagrammatic representation of the pristane injection and vehicle, Ibrutinib, L18I treatment for 2 weeks after pristane injection. **j**–**m** Representative gross illustration (**j**) and H&E staining (**l**) of lung tissue of mice treated with vehicle, Ibrutinib and L18I (50 mg/kg, i.p., once or twice a day, *n* = 8). Prevalence (**k**) and disease severity (**m**) of DAH were quantified, respectively. **n** Survival curves of mice induced by pristane treated with vehicle, Ibrutinib and L18I (50 mg/kg, i.p., once a day, *n* = 6). STS (0.3 mg/kg) was administered every two days to increase mortality of DAH in mice. **o**, **p** Proportion (**o**) and quantification (**p**) of Ly6C^hi^ and Ly6C^lo^ monocytes in lungs of pristane-induced mice treated with vehicle, Ibrutinib and L18I (50 mg/kg, i.p., once a day, *n* = 5). **q** Flow cytometry for the expression of BTK in Ly6C^hi^ and Ly6C^lo^ monocytes in lungs after 2 weeks of pristane induction (L18I: 50 mg/kg, i.p., once a day, *n* = 5). **r** Relative quantification of BTK protein level after MFI normalization is shown. Statistical analysis was performed using unpaired two-tailed Student’s *t*-test (**d**, **e**), Mann-Whitney test (**c**, **m**, **o**, **p**, **r**), log-rank test (**n**) and *t* test (**f**–**h**). *P < 0.05, **P < 0.01, ***P < 0.001, ****P < 0.0001. All data are represented as means ± SD.

Firstly, the degradation of BTK by L18I in different organs of C57BL/6 mice was examined. L18I efficiently degraded BTK protein in lymph node, as well as in lung and liver, which enabled us to investigate its effects on the treatment of autoimmune diseases (Supplementary Fig. S1d). In lupus, hyperactivation of B cells is closely correlated with the production of antibodies against autoantigens, and thus promotes the development of lupus. As a key molecule in the BCR signaling pathway, BTK is required for the efficient signaling of B-cell development, activation, differentiation and antigen presentation^4^. It has been reported that peripheral blood B cells from lupus patients have both increased BTK expression and activity^6^. Thus, BTK inhibitors targeting its kinase activity have been reported to be effective in alleviating autoimmune diseases^5^. Considering the potential advantages of PROTACs over inhibitors, particularly their ability to degrade the whole protein, which may inhibit the entire physiological functions of the target protein, we attempted to investigate the effects of L18I in murine lupus models. BM12 splenocytes were adoptively transferred to C57BL/6 mice to induce lupus-like autoimmune diseases. L18I treatment began in the second week of BM12 induction and continued for two weeks (Fig. 1a). Ibrutinib, an FDA-approved BTK inhibitor for the treatment of B-cell malignancies, served as a positive control due to its therapeutic effect in autoimmune diseases. The results showed that both L18I and Ibrutinib were effective in attenuating the BM12-induced lupus symptoms. Specifically, the amount of IgM and IgG autoantibodies, anti-double-stranded DNA (anti-dsDNA) (Fig. 1b, c) and anti-nuclear antibodies (ANA), were decreased upon treatment (Fig. 1d). The deposition of antigen–antibody immune complexes in the glomeruli was also reduced (Fig. 1e).

In biochemical studies, L18I exhibited a high degradation efficiency of BTK in primary B cells, with the half maximal effective concentration (EC_50_) being 15.36 nM (Supplementary Fig. S2a, b). In B-cells, BTK not only plays a key role in BCR signaling through its kinase activity, but also participates in TLR-related signaling pathways via its scaffolding function^4^. To directly analyze the effects of L18I on these two signaling pathways, we stimulated murine primary splenic B-cells with either F(ab’)_2_ anti-mouse IgM or LPS to activate BCR or TLR4 signaling, respectively, and detected the upregulation of B-cell activation markers including CD25, CD69 and CD86 (Supplementary Fig. S2c). When the BCR signaling pathway was activated, both L18I and Ibrutinib reduced the upregulation of activation makers. However, for the activation of TLR4 signaling pathway by LPS, Ibrutinib could only slightly reduce the upregulation of CD86, while L18I could reduce the upregulation of all three makers, indicating that L18I has a better inhibitory effect on B-cell activation after LPS stimulation than Ibrutinib (Supplementary Fig. S2d, e). These results are consistent with the reports showing that BTK’s function in boosting TLR pathway is independent of its kinase activity^7^. To validate these findings, further experiments were conducted to investigate the different inhibitory activity of Ibrutinib and L18I in B cells. RNA-seq analysis of primary B cells revealed that L18I inhibites the LPS-mediated B-cell activation through the reduction of anti-apoptosis, NF-κB, TNF, mTOR and TLR signaling pathway (Supplementary Fig. S3a). Consistent with the aforementioned results of B-cell activation makers, L18I efficiently downregulated NF-κB, TNF and TLR signaling pathway compared with Ibrutinib, with representative genes such as *Nfkb2*, *Relb*, *Akt1* and *Traf1* in these pathways (Fig. 1f–h). Meanwhile, the western blotting results of B-cell line-Ramos showed that both L18I and Ibrutinib reduced the activation levels of TLR signaling pathways, and especially L18I downregulated the phosphorylation of ERK upon the stimulation of LPS (Supplementary Fig. S3b).

In addition to B cells, BTK is also expressed in myeloid lineage cells, such as monocytes/macrophages, neutrophils and dendritic cells (DCs) (Supplementary Figs. S4 and S5), consistent with the literature reports^5^. Notably, BTK is involved in the activation and function of myeloid cells by regulating TLR signaling, FcγR signaling and NLRP3 inflammasome^5,8^, which suggest that BTK may also contribute to myeloid cells-mediated autoimmune diseases. DAH, an extremely severe complication of lupus disease with ~50% mortality, manifests as dyspnea and pulmonary infiltrates, mostly concomitant with renal diseases, elevated anti-dsDNA antibodies and hypocomplementemia^9^. However, the currently commonly used therapies for DAH, including corticosteroids and cyclophosphamide in combination with extensive supportive care^9^, have low specificity and side effects. There is an urgent need for targeted drugs for DAH. Since both B-cells and myeloid-derived monocytes/macrophages play a crucial role in the progression of DAH, as either B-cell or monocyte/macrophage-deficient mice exhibit resistance to DAH mouse model^10,11^, we speculate that BTK degraders may have a potential effect in the treatment of DAH.

DAH mouse model can be induced by pristane, a hydrocarbon oil that induces a peak of pulmonary hemorrhage in 2 weeks^10^. Following pristane induction, the mice were treated with different doses of L18I or Ibrutinib (Fig. 1i). According to the pathological feature of the lungs, we classified each pristane-induced mouse into no DAH, partial DAH and complete DAH, respectively (Supplementary Fig. S6a). Compared with the vehicle control group, L18I dramatically reduced DAH prevalence, whereas Ibrutinib only exhibited partial effectiveness (Fig. 1j, k). As a further validation, the DAH score based on lung HE staining showed that L18I reduced pulmonary hemorrhage and immune cell infiltration (Fig. 1l, m), exhibiting more effectiveness than Ibrutinib. Consistently, the weight of lungs and spleens, and total serum IgM levels were decreased in response to L18I treatment, which were independent indicators of the reduction of DAH syndromes (Supplementary Fig. S6b–e). Moreover, L18I also reduced the death rate in DAH mice (Fig. 1n). Thus, L18I exhibited certain levels of advantages over Ibrutinib in symptom relief in DAH mouse models.

Notably, pristane-induced DAH was accompanied by infiltration of a mass of immune cells into lungs, including myeloid-derived monocytes, neutrophils and DCs^10^. To further investigate cellular mechanism, we obtained lung single cells and analyzed the immune cell profiles from each pristane-induced DAH mouse (Supplementary Fig. S7). Pristane-induced mice exhibited approximately twice the cell number compared to untreated healthy mice, and L18I could reverse this phenomenon (Supplementary Fig. S8a). During the progression of DAH, the infiltration of myeloid CD11b^+^Ly6C^hi^/Ly6C^lo^ monocytes is believed to be directly proportional to the severity of DAH^10^. In our experimental system, not only the proportion but also the number of myeloid CD11b^+^Ly6C^hi^/Ly6C^lo^ monocytes were decreased after the treatment of both L18I and Ibrutinib (Fig. 1o, p). The proportion of Ly6C^hi^ monocytes in the lungs of L18I-treated mice was slightly lower than that of Ibrutinib-treated mice, which was consistent with the degree of pulmonary hemorrhage. In the literature, although B-cell-deficient or macrophage-depletion mice were resistant to pristane-induced DAH^11^, there are correlation analyses showing that the number of B cells and macrophages was independent of or even negatively correlated with the severity of DAH^10^. Here, our results showed that the proportion of B cells and macrophages in the lungs decreased after pristane induction but reversed to levels almost equivalent to those of healthy mice after L18I treatment, while Ibrutinib had no such effect (Supplementary Fig. S8b, c). Although DAH is accompanied by a large infiltration of neutrophils exhibiting a pro-inflammatory character, it has been shown that neutrophil depletion had no effect on the pulmonary hemorrhage or even worsen it^10,11^. In this perspective, it seems reasonable that L18I did not reduce neutrophils in the lungs of pristane-induced DAH mice (Supplementary Fig. S8b, c). The role of DCs in DAH remains unclear. Our results clearly showed increased DC infiltration in the lungs after pristane induction, and both L18I and Ibrutinib treatments normalized the levels of DCs in pristane-treated lungs (Supplementary Fig. S8b, c). Combining the severity of DAH and the subsets of infiltrating immune cells in the different treatment groups, we concluded that both L18I and Ibrutinib could alleviate the symptoms of DAH in mice, and that L18I appeared to be more effective than Ibrutinib in restoring a normal immune cell microenvironment in the pristane-treated mouse lung.

Since L18I can degrade BTK protein to a relatively low level in lung (Supplementary Fig. S1d), we were curious about the BTK levels in various primary immune cell subsets in pristane-treated mouse lungs administrated with L18I. Flow cytometry showed that BTK protein levels in both CD11b^+^Ly6C^hi^ and CD11b^+^Ly6C^lo^ monocytes were effectively decreased by L18I (Fig. 1q, r). Additionally, L18I can also degrade BTK protein in primary murine B cells and human THP1 monocytes (Supplementary Fig. S9). While L18I did not significantly reduce the BTK levels in DCs, macrophages and neutrophils, a decreased trend was observed (Supplementary Fig. S9b, c). These results suggested that L18I might alleviate DAH symptoms primarily by degrading BTK in monocytes, followed by B cells, DCs, macrophages and neutrophils.

Pristane-induced increased inflammatory response and secretion of inflammatory cytokines are important promoters of DAH pathology. The role of BTK in monocytes/macrophages is mainly reflected in the production of inflammatory factors. Therefore, we induced bone marrow-induced monocytes/macrophages (BMDMs) in vitro, and investigated the effects of L18I on the FcγR signaling, TLR signaling and NLRP3 inflammasome in monocytes/macrophages. The results showed that both L18I and Ibrutinib could significantly inhibit the upregulation of inflammatory cytokines after the activation of FcγR and TLR signaling in BMDMs, and L18I was somewhat superior to Ibrutinib in reducing *Tnfa* expression (Supplementary Fig. S10a, b). When the NLRP3 inflammasomes were activated, L18I could reduce the *Tnfa* and *Il1b* transcription and even IL-1β secretion, which was superior or comparable to Ibrutinib treatment (Supplementary Fig. S10c, d). Consistently, we obtained the same results in THP1 monocytes (Supplementary Fig. S10e). In general, L18I can effectively reduce the production of pro-inflammatory cytokines in monocytes/macrophages under different stimulation conditions, superior to Ibrutinib in some experimental conditions. However, it is worth noting that BTK kinase activity is also crucial in the TLR signaling of monocytes/macrophages, explaining Ibrutinib’s effect on reducing inflammatory cytokines^4,5^.

Combined with the above results, L18I can not only efficiently relieve lupus symptoms by reducing antibody secretion but also alleviate DAH by lessening the inflammatory responses. Considering the multiple side effects of Ibrutinib due to off-target effects and the emergence of new mutations^12,13^, L18I has the potential to become a safer and more effective alternative to BTK inhibitors in the treatment of autoimmune diseases. In addition, our study highlighted the advantages of BTK degrader over BTK inhibitor in alleviating TLR signal pathway activation, which is consistent with the recent finding of another BTK inhibitor, NX-2127, in disrupting BTK scaffolding function in B lymphoma^14^. Our study also demonstrates therapautic effects of L18I in monocyte-dominated diseases, which reminds us of the reported disease exacerbation in COVID-19 patients accompanied by monocyte infiltration and pro-inflammatory cytokine secretion^15^. Given the similarity to DAH, it is promising that the symptoms of pneumonia, caused by including but not limited to SARS-CoV-2, may also be alleviated by L18I.

### Supplementary information

Supplementary Materials for PROTAC for Bruton’s tyrosine kinase degradation alleviates inflammation in autoimmune diseases
