# Supplementary material for: PROTAC for Bruton’s tyrosine kinase degradation alleviates inflammation in autoimmune diseases
Source: Cell Discov. 2024 Aug 6;10:82. doi: 10.1038/s41421-024-00711-x (PMC11303405; doi:10.1038/s41421-024-00711-x)
Supplement: Supplementary file 1 — Supplementary Materials for PROTAC for Bruton’s tyrosine kinase degradation alleviates inflammation in autoimmune diseases [file 41421_2024_711_MOESM1_ESM.pdf]

Supplementary Materials for

PROTAC for Bruton's tyrosine kinase degradation alleviates inflammation in autoimmune diseases

This PDF file includes:

Materials and Methods

Supplementary Figures S1-S10

Materials and Methods

Mice

C57BL/6 (JAX664) and BM12 (JAX1162) mice were purchased from the Jackson laboratory. 6-8 week female mice were used in the experiments. Mice were maintained under specific pathogen free (SPF) conditions in the animal facility of Tsinghua University. Mouse experiments were performed in accordance with governmental and institutional guidelines for animal welfare, and approved by the Institutional Animal Care and Use Committee (IACUC) of Tsinghua University.

Cells

Jurkat cells, PC-9 cells, WM115 cells, Ramos cells, THP-1 cells, U266 cells and J558L cells were cultured in RPMI or DMEM medium supplemented with 10% fetal bovine serum (FBS), 100 U/mL penicillin/streptomycin antibiotics, non-essential amino acids, 10 mM HEPES and 50 μM β-mercaptoethanol. Primary B cells were isolated from the spleen, and cultured in complete RPMI medium. Bone marrow-derived monocytes/macrophages (BMDMs) were prepared from bone marrow cells isolated from the femurs and tibiae of wild-type C57BL/6J mice. Cells were counted and then cultured in complete RPMI medium with 30% L929 supernatant for 7 days. Adherent BMDMs were used for the following experiments.

Antibodies

Antibodies against BTK (#8547), Phospho-ERK1/2 (#4370), ERK1/2 (#9102), Phospho-Tyr1217-PLCγ2 (#3871), PLCγ2 (#3872), Phospho-Ser1248-PLCγ1 (#8713), PLCγ1 (#5690), Phospho-Thr180/Tyr182-p38 MAPK (#9211), p38 MAPK (#9212), ITK (#2380), HER/ErbB2 (#2165), GAPDH (#5174) and β-actin (#4970) were provided by Cell Signaling Technology (Danvers, MA, USA). The anti-EGFR (AF5153) antibodies were obtained from Beyotime Biotechnology (Haimen,

China). Flow cytometry related antibodies include PE anti-BTK (#558527), percp-cy5.5 anti-mouse CD25 (#101912), FITC anti-mouse CD69 (#104506), PE-cy7 anti-mouse CD86 (#105014), APC anti-mouse B220 (#103212), BV421 anti-mouse CD11b (#101236), FITC anti-mouse Ly6C (#128006), PE anti-mouse Ly6G (#127608), FITC anti-mouse CD11c (#117306), AF647 anti-mouse Siglec-F (#155520) and PE anti-mouse MHCII (#107608) were purchased from Biolegend or BD. Goat anti-mouse IgM (#1020-01), HRP goat anti-mouse IgM (#1021-05), IgG (#1033-05) were purchased from SouthernBiotech. AF488 anti-mouse IgM (#115-545-020), AF647 anti-mouse IgG (#115-605-008), F(ab')$_2$ anti-mouse IgM (#115-006-075) were purchased from Jackson ImmunoResearch.

BM12-induced lupus-like mouse model

To induce lupus-like model, splenocytes (1 x 10^7 per mouse) from age and gender-matched BM12 mice were adoptively transferred to 6-8-week wild-type C57BL/6 mice for two times on a weekly basis. Vehicle, Ibrutinib and L18I were administered daily from weeks 2 to 4, and the mice were sacrificed at week 4 for detection of autoantibodies and glomerular immune-complex deposition.

Enzyme-linked immunosorbent assay (ELISA)

For anti-dsDNA detection, 4 μg/mL dsDNA was coated on the ELISA plates with DNA-coating solution (Thermo Fisher) overnight at 4 °C. Blocked with 5% skim milk for 2 h at room temperature (RT), these plates were added gradient dilutions of mouse serum and incubated for 1.5 h at RT. Peroxidase-conjugated goat anti-mouse IgM and IgG were used for detection. These plates were washed with PBS/0.05% Tween-20 between each two steps. To detect the total IgM in pristane-induced DAH mice, 4 μg/mL goat anti-mouse IgM was coated on the ELISA plates overnight at 4 °C. The optical density was measured by ELISA plate reader (Bio-Rad). Antibody titers were

determined by the maximum dilution that is close to the two times of background OD value. Anti-nuclear antibodies (ANA) were quantified through immunofluorescence with ANA detection kit following the recommended protocols.

Immunohistochemistry for immune complexes in glomeruli

Kidneys were obtained from BM12-induced lupus mice, fixed with 4% paraformaldehyde, cryoprotected with 30% sucrose solution and frozen in O.C.T compound at −80 °C, and then sliced into 8μm sections for immunohistochemistry. Kidney slides were rehydrated and blocked, then stained with fluorophore-coupled goat anti-mouse IgM and IgG specific antibodies in staining buffer (100 mM Tris-HCl, pH 8.0, 0.3% Triton X-100) at 4 °C for 12 h. Slides were washed and mounted with ProLong Gold Anti-fade Mountant containing 4′, 6-diamidino-2-phenylindole (DAPI) (Life Technologies). Sections were imaged with the Olympus FLUOVIEW FV1000 confocal laser scanning microscope.

DAH induction and identification of DAH degree

DAH was induced by a one-time intraperitoneal injection of 0.8 ml pristane (MACKLIN) in 6-8 week wild-type C57BL/6 mice. We identified the degree of DAH after 2 weeks of pristane induction. Lungs from pristane-induced mice were divided into no DAH, partial DAH and complete DAH according to the appearance of the lungs. Lungs were fixed with 4% paraformaldehyde for more than 24 h, then embedded in paraffin and sliced into 5 μm sections for H&E staining. DAH severity was scored based on the total area of hemorrhage: 0 = no hemorrhage; 1 = 1%-20%; 2 = 21%-40%; 3 = 41%-60%; 4 = 61%-80%; 5 ⩾ 81%. Each animal was scored by two individual researchers. To increase mortality in mice with pristane-induced DAH, Staurosporine (STS, 0.3 mg/kg) was administered every two days, and death was observed daily.

L18I and Ibrutinib administration

For mouse experiments, L18I and Ibrutinib were dissolved in PBS containing 10% DMSO and 10% Cremophor EL. The vehicle group was treated with PBS containing 10% DMSO and 10% Cremophor EL. We set the healthy and vehicle group as negative and positive control, respectively. Vehicle, Ibrutinib and L18I (50 mg/kg, i.p., twice a day) were administered daily from weeks 2 to 4 after BM12-splenocytes transfer. The drugs were administered daily (50 mg/kg, i.p., once or twice a day) from 2 days to 14 days after pristane injection. For *in vitro* cell experiments, L18I and Ibrutinib were dissolved in DMSO and cells were treated with 100 nM or 200 nM L18I and Ibrutinib under different experimental conditions.

Western Blotting

To detect the off-target effects of L18I, Jurkat cells, PC-9 cells and WM115 cells were treated with L18I for 24 hours. To detect the activation of signaling pathways, Ramos cells were treated with Ibrutinib or L18I in presence or absence of LPS stimulation for 24 hours. To detect the BTK expression and degradation within different immune cell lines including Ramos, THP-1, Jurkat, U266 and J558L cells, these cells were treated with different concentrations of L18I for 24 hours. The collected cells were lysed using RIPA buffer containing protease/phosphatase inhibitor cocktail (Cat No. 04693124001, Roche, Mannhein, Baden-Wuerttemberg, Germany). The BCA assay was carried out to quantify protein (Cat No. 23227, Thermo), and equivalent amounts of protein samples were separated by SDS-PAGE contained 4-12% acrylamide (Cat No. NPO335BOX, Invitrogen, Carlsbad, CA). After the protein was transferred to a PVDF membrane, the proper primary and secondary antibodies were probed and used for chemiluminescence detection. To detect the BTK degradation in mice and different primary cells, Vehicle and L18I (50 mg/kg, i.p., twice a day) were

administered daily for 4 days. Different tissues were harvested from animals and flash frozen in liquid nitrogen. Specifically, small pieces (10–20 mg) of the collected tissues were lysed with RIPA lysis buffer containing 1% PMSF and 1% protease inhibitor on ice for 1 h. Primary mouse monocytes, T cells, and B cells were purified from peripheral blood and spleen, and were lysed using RIPA buffer containing protease/phosphatase inhibitor cocktail. Protein samples were collected for concentration determination and further analysis as described previously

Flow cytometry of lung immune cells

Fresh lungs were obtained and chopped with scissors. The digestion solution was prepared by adding 0.5 mg/mL collagenase IV and 0.1 mg/mL DNase I to the incomplete RPMI medium. Each lung was digested with 5 ml of digestion solution on a shaker at 37 °C for 1.5 h, gently pressed with a syringe rubber plug, and then passed through a 70 μm screen. The digested cells were collected, washed once with PBS and lysed with red blood lying buffer, and the lung single-cell suspension was obtained. After blocking with 2.4G2 on ice for 15 min, the cells were stained with CD11b, Ly6G, Ly6C, CD11c, Siglec-F, MHCII and B220 antibodies for 20 min at 4℃, and washed once with PBS. Then the dead and alive cells were distinguished by 7-AAD antibody, and the cells were directly detected by Fortessa flow cytometer (BD) by adding appropriate amount of PBS. In order to detect the levels of BTK protein, cells were fixed and permeabilized using Fix/Perm kit for intracellular staining of BTK antibody, and the fluorescence intensity of BTK was detected by Fortessa flow cytometer (BD). We use the MFI of BTK in the NC group without BTK antibody as the background. The BTK protein content was characterized by subtracting the background MFI value from the BTK-MFI of vehicle group and L18I group, respectively. Then, with the average value of vehicle group normalized as 1, the relative amount of BTK in each mouse was calculated,

and the degradation ratio of BTK protein could be calculated.

Stimulation of primary B cells *in vitro*

Primary B cells were isolated from murine spleen and the purity of B cells was confirmed by flow cytometry. 2 x 10^5 cells were cultured in 96-well plates, treated with DMSO, Ibrutinib and L18I (100 or 200 nM) and then stimulated by $F(ab')_2$ anti-mouse IgM (10 μg/mL) and LPS (1 μg/mL). After 24 hours of stimulation, the expression of activation makers CD25, CD69 and CD86 on the surface of primary B cells was analyzed by flow cytometry.

RNA-seq analysis

1 x 10^6 primary B cells were cultured in 24-well plates, treated with the DMSO, Ibrutinib and L18I (200 nM) and then stimulated by LPS (1 μg/mL) for 12 hours, with the control group without LPS stimulation. Then total RNA was purified by trizol method, and the RNA integrity was confirmed by 2100 Bioanalyzer (Agilent Technologies Santa Clara, CA, USA) and Qubit Fluorometer (Invitrogen). Sequencing was performed by Illumina NovaSeq sequencer (Illumina, San Diego, CA, USA) according to the manufacturer's instructions, and data processing and analyzing were performed by CapitalBio Technology (Beijing, China).

Stimulation of FcγR signaling, TLR signaling and NLRP3 inflammasome

To stimulate the FcγR signaling of BMDMs, 1 μg/mL mouse IgG was incubated in a 24-well plate at 4 °C for 24 h. The induced BMDMs were digested and counted, pretreated with DMSO, Ibrutinib (100 nM) or L18I (100 nM) and placed in the well coated with mouse IgG for 24 h. To stimulate the TLR signaling, BMDMs were pretreated with DMSO, Ibrutinib or L18I for 4 hours and then stimulated by 0.5 μg/mL LPS for 20 h. BMDMs or THP1 cells, pretreated with DMSO, Ibrutinib or L18I for 2 h, were primed with 50 ng/mL, 100 ng/mL or 200 ng/mL LPS for 3 h at 37°C and then

stimulated with 10 μM Nigericin (NI) for 1h. After stimulation, cells were collected to quantify the *Tnfa*, *Il1b* and *Il6* expression, and supernatants were collected to determine the IL-1β levels by ELISA using human or mouse IL-1β detection kit.

RT-qPCR

Total RNA of stimulated cells was obtained by RNA extraction kit, and was reversed into cDNA by reverse transcription kit. qPCR was performed using SYBR in Bio-Rad CFX96 Touch for 96-well plate. Primers used in this experiment were mouse-*Tnfa* (forward: GAACTGGCAGAAGAGGCACT; reverse: AGGGTCTGGGCCATAGAACT), mouse-*Il1b* (forward: GCCCATCCTCTGTGACTCAT; reverse: AGGCCACAGGTATTTTGTCG) and mouse-*Il6* (forward: TCTGCAAGAGACTTCCATCCAGTTGC; reverse: AGCCTCCGACTTGTGAAGTGGT). Gene expression was normalized to mouse-*Actin* (forward: GTGACGTTGACATCCGTAAAGA; reverse: GCCGGACTCATCGTACTCC), and was determined by using the ΔΔCt method.

Statistical analysis

Statistical analyses were performed using GraphPad Prism 5.0 software and displayed in the form of mean ± SD. Flow cytometry analysis was performed with Flowjo software. Images were processed and analyzed using Image J software. Differences between 2 groups were analyzed by unpaired Student's t test, unpaired Mann-Whitney test, log-rank test, and paired Wilcoxon test. All tests were 2-tailed unless stated otherwise. P values are indicated by *$P < 0.05$, **$P < 0.01$, ***$P < 0.001$, ****$P < 0.0001$, ns: no significance.

Figure S1

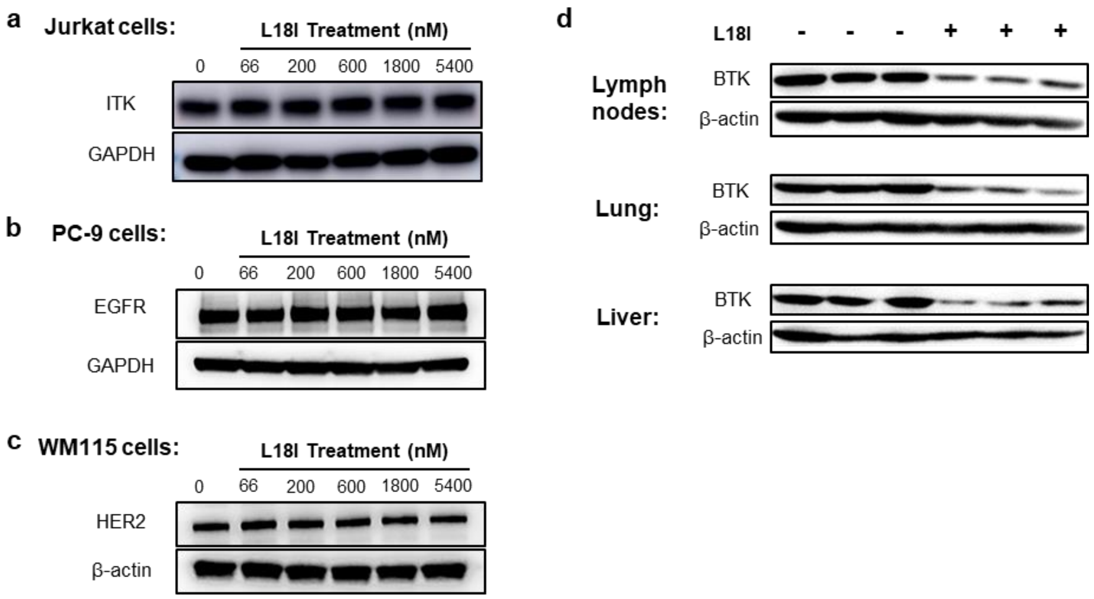

Fig. S1 L18I had no off-target effect in cell lines and effectively degraded BTK protein *in vivo*.

a-c Human T cell acute lymphoblastic leukemia Jurkat cells, human lung cancer PC-9 cells and human primary melanoma WM115 cells were treated with different concentrations of L18I for 24 hours. The expression of ITK (**a**), EGFR (**b**) and HER2 (**c**) were analyzed by Western Blotting analysis, respectively. GAPDH or β-actin was showed as loading control. **d** Immunoblotting for BTK in various organs/tissues of mice, including lymph nodes, lung and liver, given L18I (50 mg/kg, i.p., twice a day) for 4 days (n=3).

Figure S2

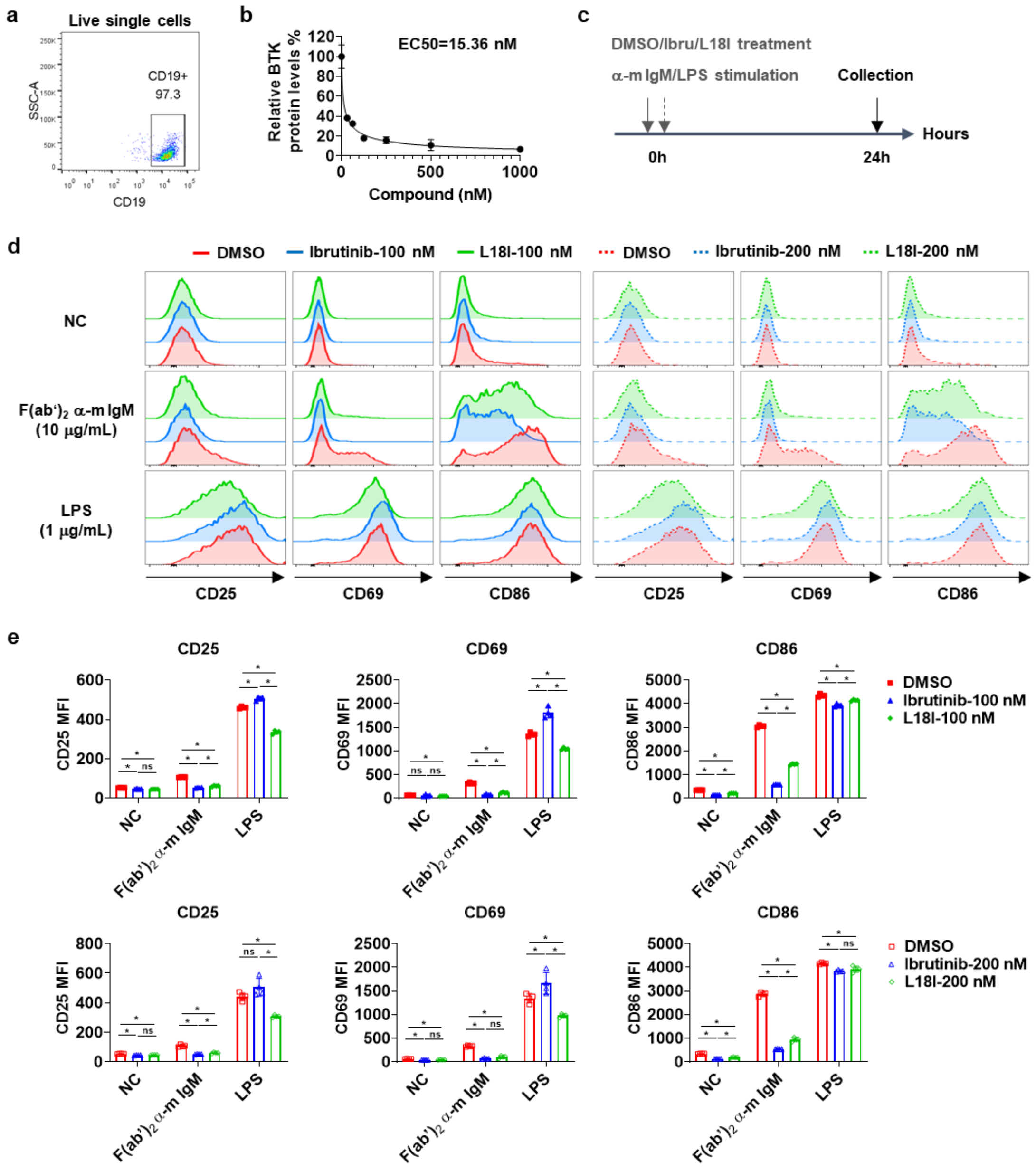

Fig. S2 L18I reduced B cell activation triggered by BCR and TLR4 stimulation. a Purity of mouse spleen primary B cells by magnetic bead negative sorting. **b** Flow cytometry analysis for relative BTK protein levels in primary B cells treated with the indicated concentrations of L18I for 24 h *in vitro*. The EC50 was calculated by Graphpad. **c** Schematic diagram of primary B cells treated with DMSO, Ibrutinib and L18I (100 or 200 nM) stimulated by F(ab')$_2$ α-m IgM (10 μg/mL) and LPS (1 μg/mL) *in vitro*. **d** Flow cytometry analysis for expression of activation makers CD25, CD69

and CD86 on the surface of primary B cells after treatment and stimulation for 24 h *in vitro*. The MFI of CD25, CD69 and CD86 was quantified in **e** (n=4). Statistical analysis in **e** was performed using unpaired two-tailed Mann-Whitney test. **P* < 0.05, ns: no significance.

Figure S3

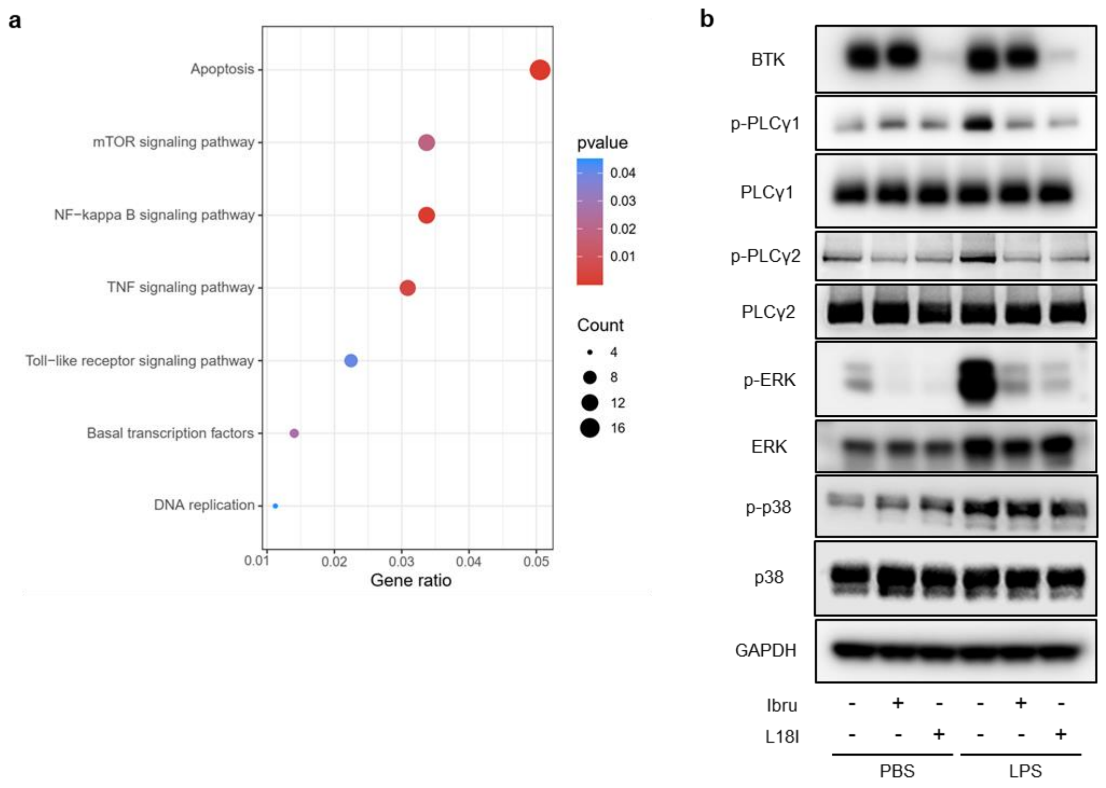

Fig. S3 L18I reduced activation levels of TLR and NF-κB signaling pathways. a Primary B cells from mouse spleen were treated with DMSO, Ibrutinib and L18I (200 nM) and stimulated by LPS (1 μg/mL) for 12 h *in vitro*. Compared with LPS alone treatment group, KEGG enrichment of down-regulated genes of L18I treatment group in presence of LPS stimulation was analyzed using RNA sequencing data. **b** The phosphorylation levels of TLR signaling pathway were detected by Western Blotting in Ibrutinib or L18I (100 nM) treated Ramos cells under LPS stimulation for 24 hours.

Figure S4

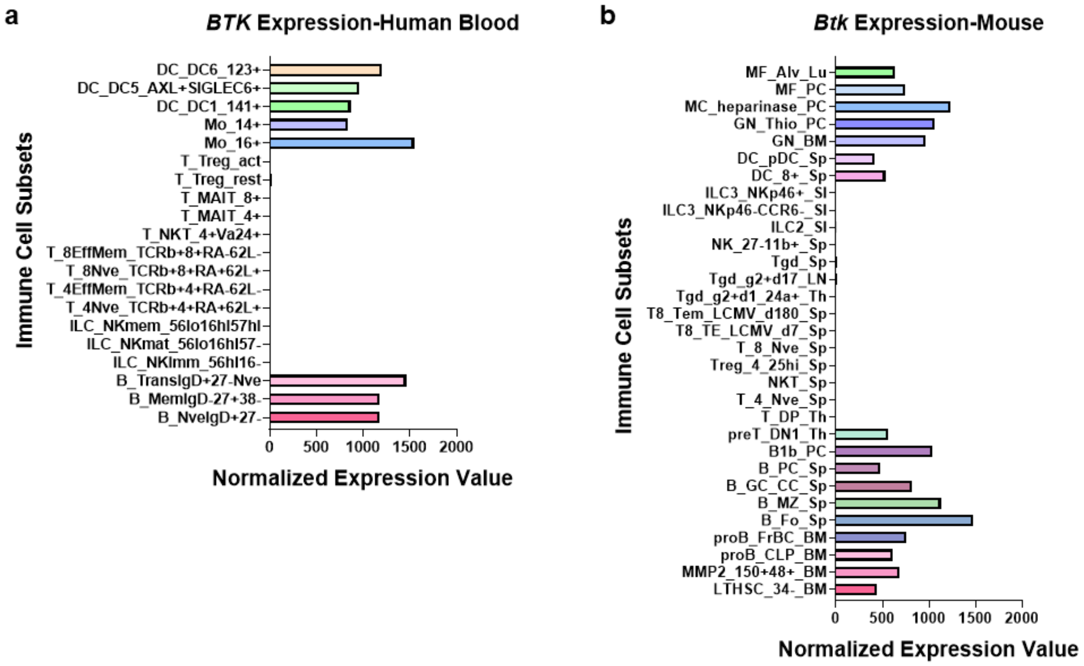

Fig. S4 *BTK/Btk* is highly expressed in B cells and myeloid cells. a, b Data of *BTK/Btk* expression in humans and mice were obtained from Immgen Databrowsers (https://www.immgen.org/). mRNA sequencing of various immune cells isolated from human blood PBMC (peripheral blood mononuclear cell) (**a**) and different tissues of mice (**b**). All expression value is normalized by DESeq2.

Figure S5

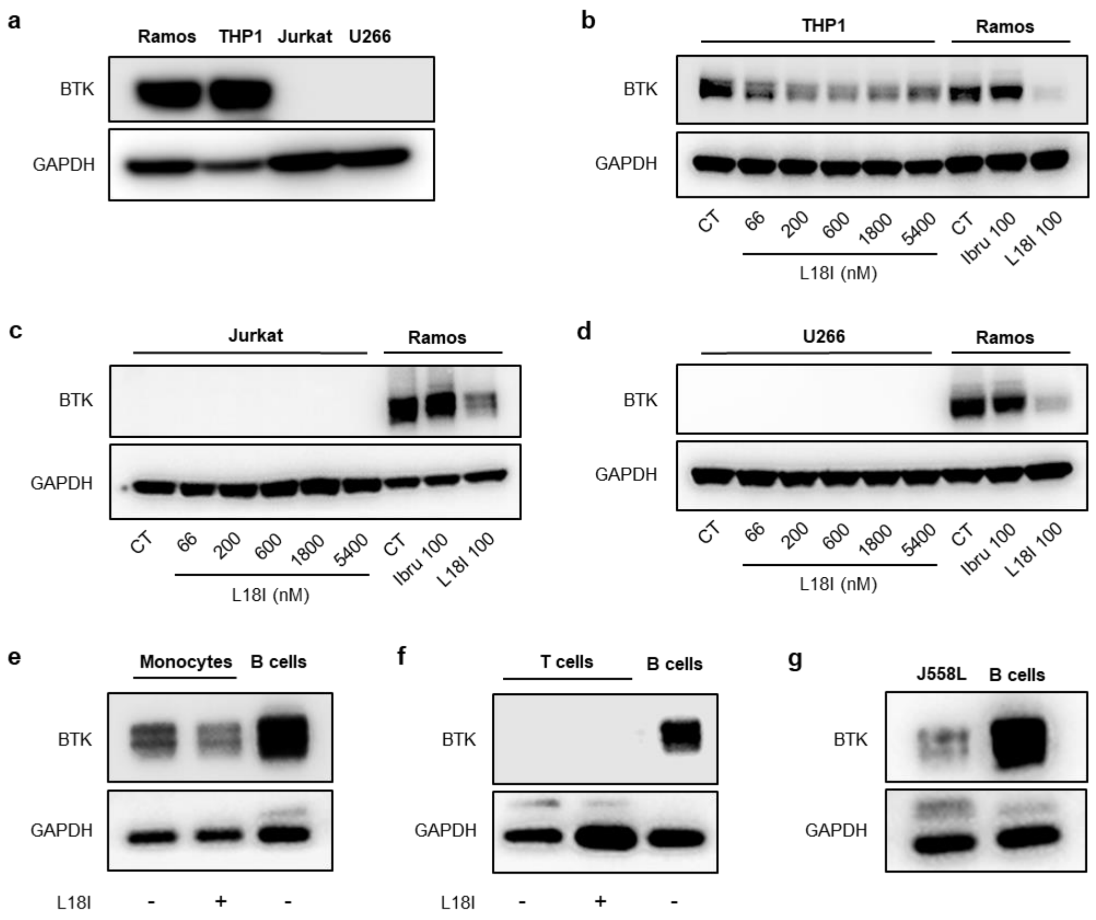

Fig. S5 Effects of BTK degrader-L18I on BTK protein within different immune cell lines and primary cells. a The expression level of BTK protein in different hematopoietic cells, including B lymphocyte cell Ramos, monocytic cell THP-1, T lymphocyte cell Jurkat and mature plasma cell U266. **b-d** THP-1 cells (**b**), Jurkat cells (**c**), U266 cells (**d**), and Ramos cells were treated with different concentrations of L18I for 24 hours, and the degradation of BTK protein was detected by Western Blotting. GAPDH is shown as a loading control. **e, f** The C57BL/6 mice were treated with L18I (50 mg/kg, i.p., twice a day) for 4 days. Primary mouse monocytes (**e**), T cells (**f**), and B cells were purified from peripheral blood and spleen. Whole cell extracts were probed by Western Blotting for BTK. GAPDH is shown as a loading control. **g** The mouse mature plasma cell line J558L cells and primary mouse B cells were harvested and subjected to Western Blotting analysis

to detect the BTK expression.

Figure S6

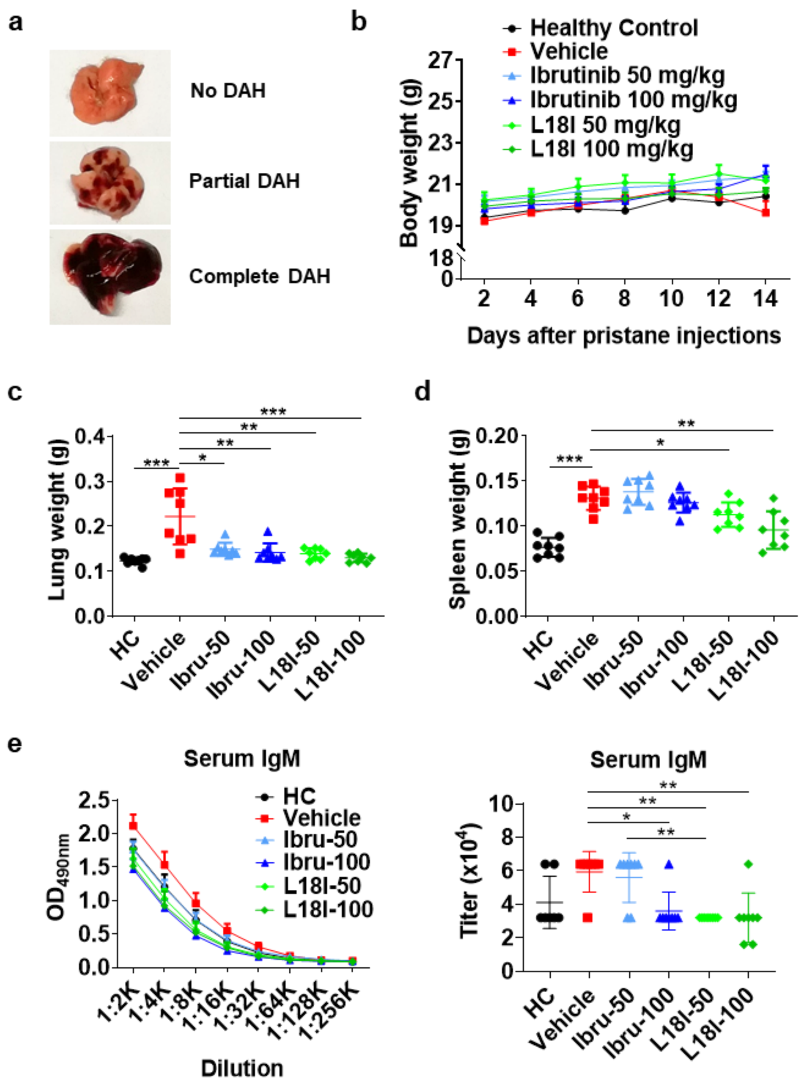

Fig. S6 Pristane-induced DAH mice showed reduced inflammation after L18I treatment. a Gross pathology of lungs in mice was classified into no DAH, partial DAH, and complete DAH. **b** Body weight of mice treated with Vehicle, Ibrutinib and L18I from 2 to 14 days after pristane induction. **c, d** Weight of lungs (**c**) and spleens (**d**) of mice treated with Vehicle, Ibrutinib and L18I (50 mg/kg, i.p., once or twice a day, n=8) after pristane induction. **e** levels of serum total IgM in pristane-induced DAH mice for 2 weeks with Vehicle, Ibrutinib and L18I treatment. Statistical analysis was performed using unpaired two-tailed Mann-Whitney test. **P* < 0.05, ***P* < 0.01, ****P* < 0.001.

Figure S7

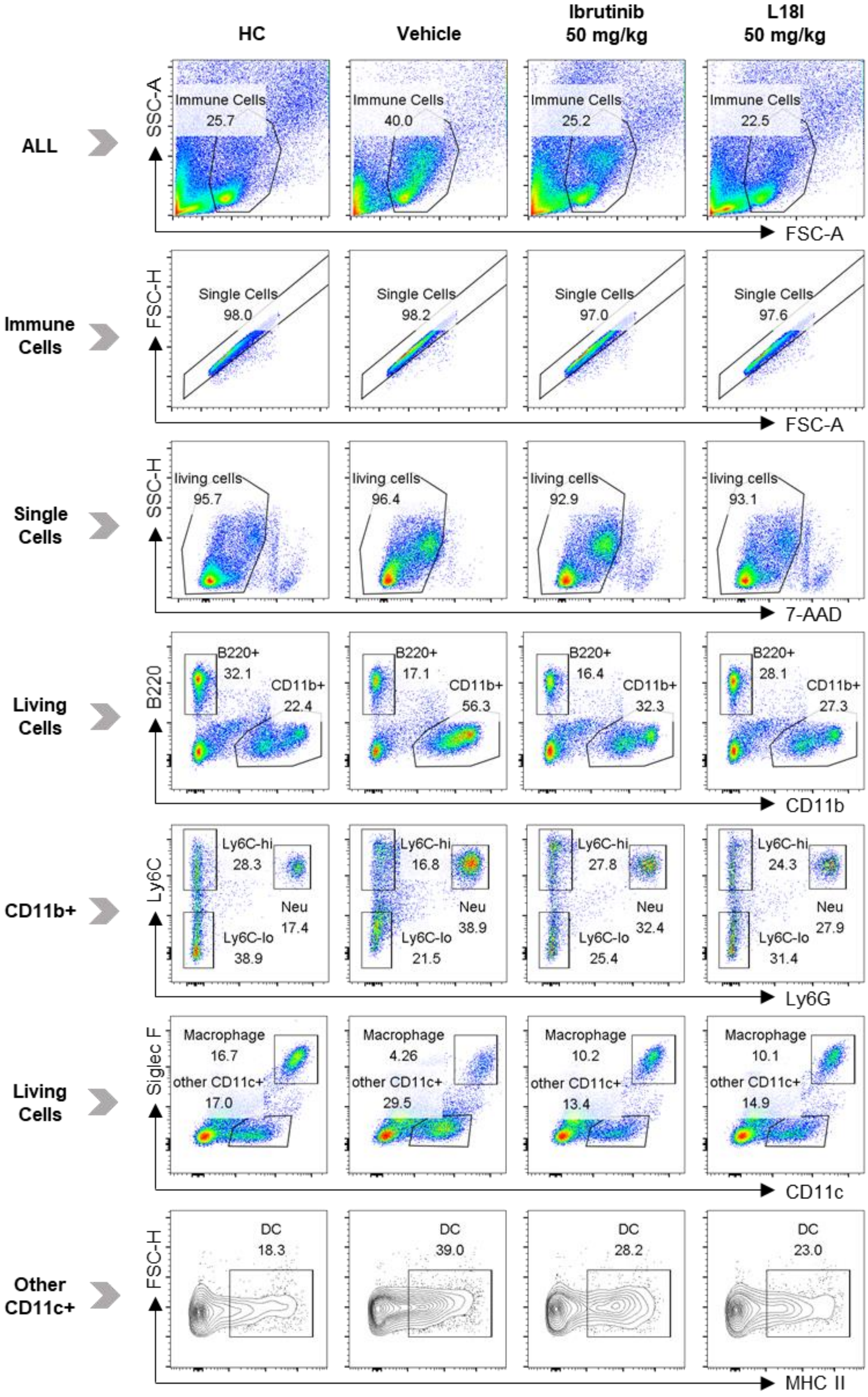

Fig. S7 Gating strategy for flow cytometry analysis of lung immune cells from pristane-induced DAH mice treated with Vehicle, Ibrutinib and L18I. B cells ($B220^{+}$), $Ly6C^{hi}$ monocytes

($CD11b^+Ly6C^{hi}Ly6G^-$), $Ly6C^{lo}$ monocytes ($CD11b^+Ly6C^{lo}Ly6G^-$), Neutrophils ($CD11b^+Ly6G^+$), Macrophages ($CD11c^+SiglacF^+$), DCs ($CD11c^+SiglacF^-MHCII^+$) in lungs were analyzed. Ibrutinib and L18I: 50 mg/kg, i.p., once a day.

Figure S8

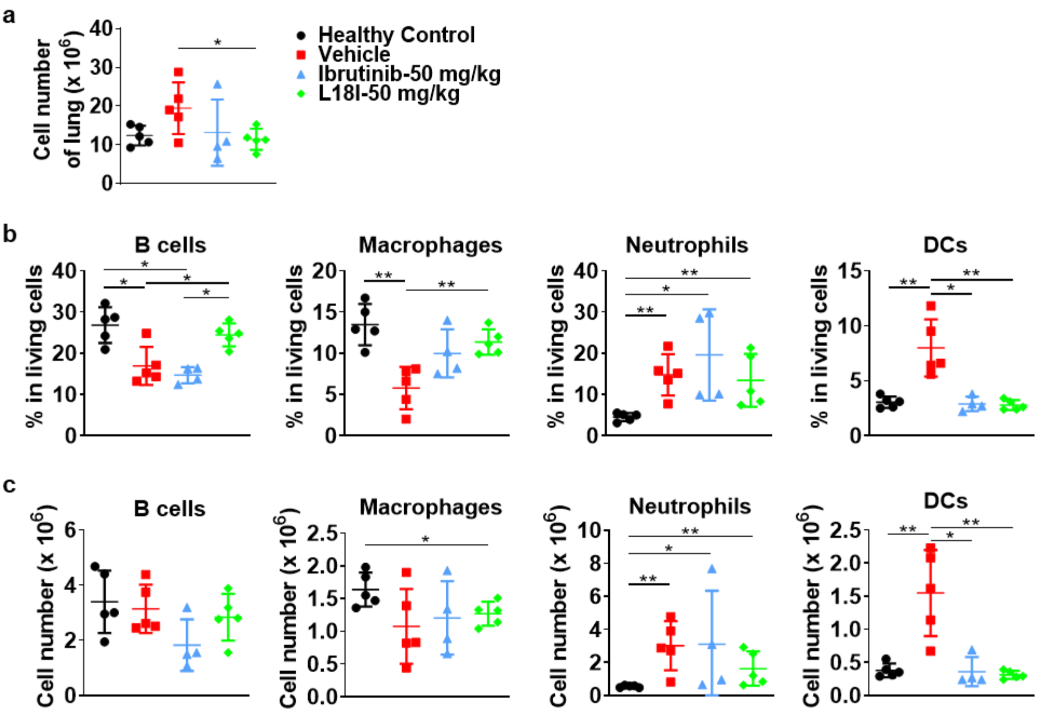

Fig. S8 Analysis of various lung immune cells from pristane-induced DAH mice. a The number of lung cells in pristane-induced mice treated with Vehicle, Ibrutinib and L18I (50 mg/kg, i.p., once a day, n=5). **b, c** Proportion (**b**) and quantification (**c**) of B cells, Macrophages, Neutrophils and DCs in lungs of pristane-induced mice. Statistical analysis was performed using unpaired two-tailed Mann-Whitney test. *$P < 0.05$, **$P < 0.01$.

Figure S9

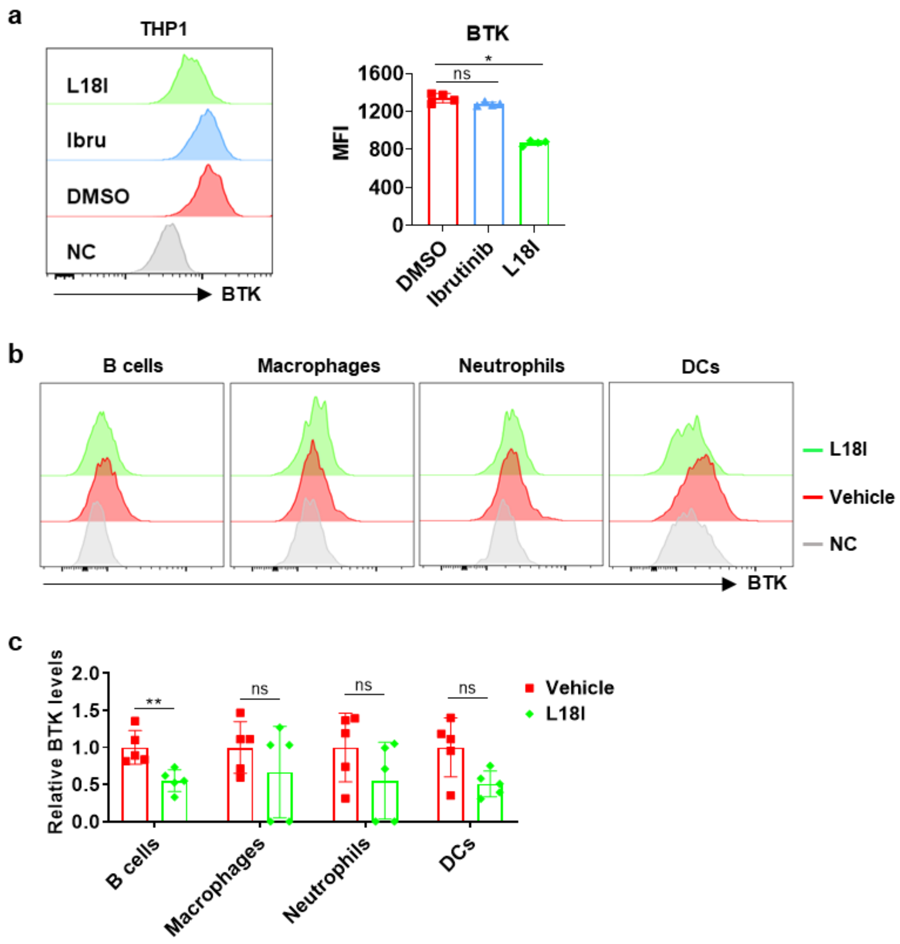

Fig. S9 Levels of BTK protein in THP1 cells and various immune cells in lungs of pristane-induced DAH mice. a Flow cytometry analysis and MFI statistical graph of BTK protein levels in THP1 cell line treated with DMSO, Ibrutinib and L18I (100 nM) for 24 h. **b** Flow cytometry analysis of BTK protein levels in B cells, Macrophages, Neutrophils and DCs in lungs of pristane-induced mice with Vehicle and L18I treatment (50 mg/kg, i.p., once a day, n=5). **c** The relative quantification of BTK protein level in different cell types after MFI normalization. Statistical analysis was performed using unpaired two-tailed Mann-Whitney test. *$P < 0.05$, **$P < 0.01$, ns: no significance.

Figure S10

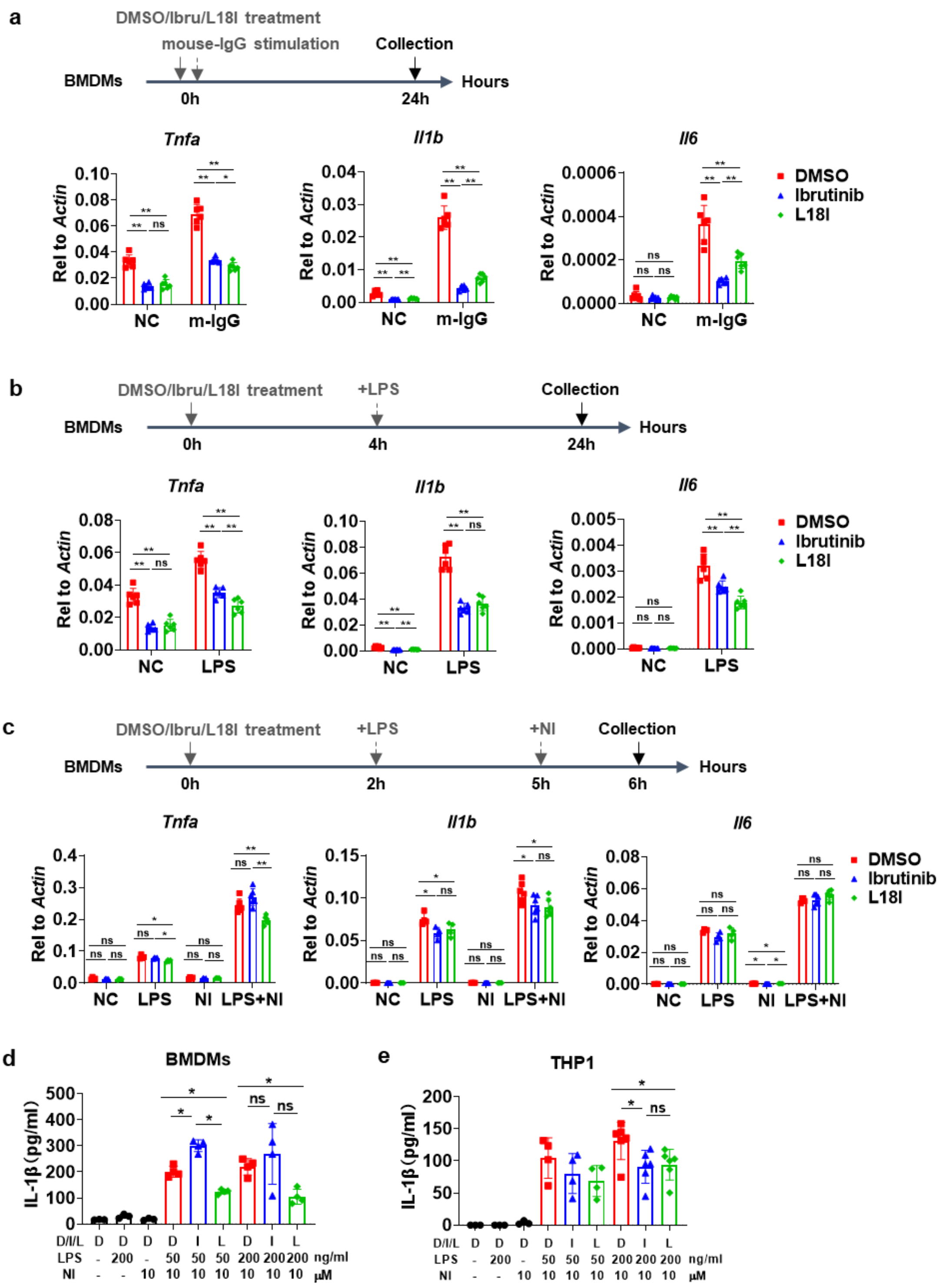

Fig. S10 L18I reduced the expression and secretion of proinflammatory cytokines in monocyte/macrophage cells. a-c The expression levels of *Tnfa*, *Il1b* and *Il6* in BMDMs after activation of FcγR signaling (**a**), TLR pathway (**b**) and NLRP3 inflammasome (**c**) (n=6).

DMSO/Ibrutinib/L18I (100 nM) treatment and various stimuli processing time patterns are shown. The concentrations of different stimuli were: **a** mouse-IgG (1 μg/mL), **b** LPS (0.5 μg/mL), **c** LPS (100 ng/mL), Nigericin (NI, 10 μM). **d, e** ELISA of IL-1β in supernatants of BMDMs (**d**) and THP1 cells (**e**) that were pretreated with DMSO/Ibrutinib/L18I (100 nM) for 2 h, primed with different concentrations of LPS for 3 h and then stimulated with 10 μM NI for 1 h. Statistical analysis was performed using unpaired two-tailed Mann-Whitney test (**a, b, c**) and paired Wilcoxon test (**d, e**). $^{*}P < 0.05$, $^{**}P < 0.01$, ns: no significance.
